# Comparison of postoperative analgesic effects in response to either dexamethasone or dexmedetomidine as local anesthetic adjuvants: a systematic review and meta-analysis of randomized controlled trials

**DOI:** 10.1007/s00540-021-02895-y

**Published:** 2021-01-30

**Authors:** Zhen-Guo Song, Shen-Yue Pang, Gui-Yue Wang, Zhao Zhang

**Affiliations:** Department of Anesthesiology, Key Laboratory of Cancer Prevention and Therapy, Tianjin’ Clinical Research Center for Cancer, Tianjin Medical University Cancer Institute and Hospital, National Clinical Research Center for Cancer, West Huanhu Road, Tiyuan, Hexi District Tianjin, People’s Republic of China

**Keywords:** Dexamethasone, Dexmedetomidine, Analgesia, Adjuvants, Nerve block

## Abstract

This review compares the effects of peripheral dexamethasone and dexmedetomidine on postoperative analgesia. We included six randomized controlled trials (354 patients) through a systematic literature search. We found that analgesia duration was comparable between dexamethasone and dexmedetomidine (58.59 min, 95% CI (confidence interval), −  66.13, 183.31 min) with extreme heterogeneity. Secondary outcome was also compared and no significant difference was observed in sensory block onset and duration and motor block duration and also for postoperative nausea and vomiting. It is noteworthy that dexamethasone reduced analgesic consumption (fentanyl) by 29.12 mcg compared with dexmedetomidine. We performed subgroup analyses and found no significant difference between the following: (1) lidocaine vs ropivacaine (*P* = 0.28), (2) nerve block vs nerve block + general anesthesia (*P* = 0.47), and (3) upper limb surgery vs thoracoscopic pneumonectomy (*P* = 0.27). We applied trial sequential analysis to assess the risks of type I and II errors and concluded that the meta-analysis was insufficiently powered to answer the clinical question, and further analysis is needed to establish which adjuvant is better. In conclusion, we believe that existing research indicates that dexamethasone and dexmedetomidine have equivalent analgesic effects in peripheral nerve blocks.

**Supplementary Information:**

The online version contains supplementary material available at 10.1007/s00540-021-02895-y.

## Introduction

Nerve blocks have been widely used for postoperative pain control in recent years, but the analgesic duration of local anesthetics is time-limited. With the development of multimodal analgesia, there are on-going studies to prolong the time of analgesic. One area of focus has been the addition of adjuvant medications to local anesthetics. Medications that have been previously investigated include opioids, clonidine, buprenorphine, dexmedetomidine, and dexamethasone [1].

Dexamethasone has been evaluated as an adjuvant either peripherally or intravenously [2]. A meta-analysis has confirmed that peripheral dexamethasone with local anesthetics prolongs the analgesic duration of the brachial plexus block [3]. The mechanism of action may involve suppressing transmission in thin unmyelinated C-fibers [4], a local vasoconstrictive effect [5], and anti-inflammatory actions [6]. Dexmedetomidine, an α_2_ adrenoreceptor, has also been found to prolong loco-regional analgesia in studies in vivo and in vitro [7, 8]. An in vivo study of a peripheral nerve block in rats found that the analgesic effect of dexmedetomidine is related to the block of hyperpolarization-activated cations [9]. Most published studies compared dexmedetomidine or dexamethasone as local anesthetic adjuvants with placebo [10, 11], and concluded that both prolong analgesia time. However, they also added their own corresponding adverse reactions: bradycardia, hypotension, and excessive sedation caused by dexmedetomidine [7, 12], whereas dexamethasone increased glucose concentration [13, 14]. Therefore, intuitive evidence is needed to compare the benefit-to-risk ratio of the two adjuvants. The objective of this systematic review and meta-analysis is to assess the effect of dexmedetomidine compared with dexamethasone peripherally on postoperative pain outcomes in patients undergoing surgery under regional or combined regional and general anesthesia.

## Materials and methods

### Search strategy and selection criteria

We followed the Preferred Reporting Items for Systematic Reviews and Meta-Analyses (PRISMA) statement guidelines [15] for the preparation of this review. Randomized controlled trials examining the effect of dexmedetomidine and dexamethasone on the duration of the block after a single-shot nerve block were evaluated using a predefined protocol (Supplemental Digital Content 1. PRISMA NMA Checklist). The review was registered on PROSPERO with the registration number CRD42020202582.

We performed a systematic electronic literature search in the databases PubMed (https://pubmed.ncbi.nlm.nih.gov/), Embase (http://www.embase.com/), the Cochrane library (https://www.cochranelibrary.com/), and Web of Science (http://apps.webofknowledge.com/) without time limits. Two authors independently screened articles to determine their qualifications. EndNote was used to manage eligible studies. We mainly used the combination of subject words and free words in the search. The exact search strategies for different databases are described in Table 1. Our search was limited to randomized trials published in the English language. Trials that are unpublished or in progress were not included.

**Table 1:** Search strategy

Search strategy for PUBMED (38)
#1. ((((((((((((((((((((regional anaesthesia) OR (Conduction Anesthesia)) OR (Anesthesia, Regional)) OR (Regional Anesthesia)) OR (nerve block)) OR (Block, Nerve)) OR (Blocks, Nerve)) OR (Nerve Blocks)) OR (Nerve Blockade)) OR (Blockade, Nerve)) OR (Blockades, Nerve)) OR (Nerve Blockades)) OR (Chemical Neurolysis)) OR (Chemical Neurolyses)) OR (Neurolyses, Chemical)) OR (Neurolysis, Chemical)) OR (Chemodenervation)) OR (Chemodenervations)) OR (peripheral block))
#2. (((((((((((Dexamethasone) OR (Methylfluorprednisolone)) OR (Hexadecadrol)) OR (Decameth)) OR (Decaspray)) OR (Dexasone)) OR (Hexadrol)) OR (Oradexon)) OR (Glucocorticoid)) OR (cortison)) OR (corticosteroid)))
#3. ((((((((Medetomidine) OR (Levomedetomidine)) OR (Medetomidine Hydrochloride)) OR (Hydrochloride, Medetomidine)) OR (Dexmedetomidine)) OR (Precedex)) OR (Dexmedetomidine Hydrochloride)) OR (Hydrochloride, Dexmedetomidine))
#4. #1 AND #2 AND #3
Search strategy for The Cochrane Library (10)
#1 MeSH descriptor: [Nerve Block] explode all trees
#2 (“Block, Nerve” or “Blocks, Nerve” or “Nerve Blocks” or “Nerve Blockade” or “Blockade, Nerve” or” Blockades, Nerve” or “Nerve Blockades” or “Chemical Neurolysis” or “Chemical Neurolyses” or “Neurolyses, Chemical” or “Neurolysis, Chemical”):ti,ab,kw
#3 #1 or #2
#4 MeSH descriptor: [dexamethasone] explode all trees
#5 (Methylfluorprednisolone or Hexadecadrol or Decameth or Decaspray or Dexasone or Dexpak or Maxidex or Millicorten or Oradexon or Decaject or Hexadrol):ti,ab,kw
#6 #4 or #5
#7 MeSH descriptor: [dexmedetomidine] explode all trees
#8 (Levomedetomidine or “Hydrochloride, Medetomidine” or “Medetomidine Hydrochloride” or Medetomidine or Precedex or “Dexmedetomidine Hydrochloride” or “Hydrochloride, Dexmedetomidine”):ti,ab,kw
#9 #7 or #8
#10 #3 and #6 and #9
Search strategy for Web of Science (46)
#1 TS = (regional anaesthesia OR Conduction Anesthesia OR Anesthesia, Regional OR Regional Anesthesia OR nerve block OR Block, Nerve OR Blocks, Nerve OR Nerve Blocks OR Nerve Blockade OR Blockade, Nerve OR Blockades, Nerve OR Nerve Blockades OR Chemical Neurolysis OR Chemical Neurolyses OR Neurolyses, Chemical OR Neurolysis, Chemical OR Chemodenervation OR Chemodenervations OR peripheral block)
#2 TS = (Dexamethasone OR Methylfluorprednisolone OR Hexadecadrol OR Decameth OR Decaspray OR Dexasone OR Hexadrol OR Oradexon OR Glucocorticoid OR cortison OR corticosteroid)
#3 TS = (Medetomidine OR Levomedetomidine OR Medetomidine Hydrochloride OR Hydrochloride, Medetomidine OR Dexmedetomidine OR Precedex OR Dexmedetomidine Hydrochloride OR Hydrochloride, Dexmedetomidine)
#4 #1 and #2 and #3
Search strategy for EMBASE (114)
1. (‘dexmedetomidine’/exp OR ‘precedex’:ab,ti OR ‘dexmedetomidine hydrochloride’:ab,ti OR ‘hydrochloride, dexmedetomidine’:ab,ti OR ‘medetomidine’:ab,ti OR ‘levomedetomidine’:ab,ti OR ‘medetomidine hydrochloride’:ab,ti OR ‘hydrochloride, medetomidine’:ab,ti)
2. (‘dexamethasone’/exp OR ‘methylfluorprednisolone’:ab,ti OR ‘hexadecadrol’:ab,ti OR ‘decameth’:ab,ti OR ‘decaspray’:ab,ti OR ‘dexasone’:ab,ti OR ‘dexpak’:ab,ti OR ‘oradexon’:ab,ti OR ‘decaject’:ab,ti OR ‘hexadrol’:ab,ti OR ‘glucocorticoids’:ab,ti OR ‘glucocorticoid’:ab,ti OR‘ cortison’:ab,ti OR ‘corticosteroids’:ab,ti OR ‘corticoids’:ab,ti)
3. (‘nerve block’/exp OR ‘block, nerve’:ab,ti OR ‘blocks, nerve’:ab,ti OR ‘nerve blocks’:ab,ti OR ‘nerve blockade’:ab,ti OR ‘blockade, nerve’:ab,ti OR ‘blockades, nerve’:ab,ti OR ‘nerve blockades’:ab,ti OR ‘chemical neurolysis’:ab,ti OR ‘chemical neurolyses’:ab,ti OR ‘anesthesia, regional’:ab,ti OR ‘peripheral block’:ab,ti OR ‘regional anesthesia’:ab,ti)
4. 1 and 2 and 3

### Inclusion and exclusion criteria

We included randomized controlled trials assessing the duration of analgesia after adding peripheral dexmedetomidine or dexamethasone as an adjuvant to local anesthetics. We performed inclusion criteria according to PICO [16].

Patients: adults undergoing surgery with peripheral nerve block alone or combined with general anesthesia.

Intervention: addition of dexamethasone to local anesthetic for perioperative analgesia.

Comparison: addition of dexmedetomidine to local anesthetic for perioperative analgesia.

Outcome: duration of analgesia, sensory block onset and duration time, motor block onset and duration time, analgesic consumption, and adverse effects.

Patients aged under 18 years and animal studies were excluded. Similarly, we also excluded observational cohort studies, case–control studies, and reviews.

### Data collection and presentation

Two authors extracted data independently. Disagreements were resolved by discussion until a consensus was reached or by consulting a third author. We selected the duration of analgesia as the primary outcome, while sensory block onset and duration time, motor block onset and duration time, analgesic consumption, and adverse effects were secondary outcomes. The duration of analgesia was defined as the time from onset of adequate sensory block to the time that the patient first requested analgesic medication. We also defined sensory and motor block onset as the time interval between the end of local anesthetic injection and the loss of pinprick sensation or motor function. Sensory and motor block duration were considered as the time interval between a successful block and the complete reappearance of all the senses and recovery of motor function. Analgesic consumption was defined as postoperative fentanyl consumption. We also retrieved perioperative adverse effects such as bradycardia, hypotension, dizziness, postoperative nausea and vomiting, Horner’s syndrome, hoarseness of voice, and hyperglycemia.

### Assessment of bias risks

Two reviews independently assessed the quality of the selected studies according to the Cochrane collaboration's tool [17] for randomized controlled trials. We used the Review Manager 5.3 Risk of Bias tool to analyze the methodological quality of the studies. This tool allows for an assessment of the risks of selection bias (random sequence generation, allocation concealment), performance bias (blinding of participants and personnel), detection bias (blinding of outcome assessment), attrition bias (incomplete outcome data), reporting bias (selective reporting), and other bias.

### Meta-analyses

We decided to perform meta-analyses when at least two studies were identified. Review Manager (RevMan, version 5.3) Copenhagen: The Nordic Cochrane Centre, The Cochrane Collaboration, 2014 was used for the meta-analysis. Dichotomous and continuous outcomes were analyzed using random-effects modeling. The risk ratios and 95% confidence intervals (CIs) are reported for dichotomous outcomes, while the mean difference and 95% CI are reported for continuous outcomes. The heterogeneity of the eligible studies was measured using the *I*^2^ test [18], we explored the sources of heterogeneity of the primary outcome by subgroup analysis or sensitivity.

### Subgroup analysis

We grouped the included studies and performed subgroup analysis three times according to the types of local anesthetics, methods of anesthesia, and type of surgery. The specific classification is as follows: (1) lidocaine vs ropivacaine, (2) nerve block vs nerve block + general anesthesia, and (3) upper limb surgery vs thoracoscopic pneumonectomy.

### Sensitivity analysis

Some studies have certain characteristics, for example, the methodological quality of several studies is low or the sample is small, we can judge whether these characteristics have affected the conclusion through sensitivity analysis, that is, by adding or removing these studies and observing the consistency of the meta-analysis.

### Trial sequential analysis

When the number of trials included in a meta-analysis is small with an insufficient sample size, random errors may lead to erroneous results [19, 20]. Trial sequential analysis is a statistical approach that combines multiple techniques, it quantifies the required evidence and provides specific values for the required information size. Results are presented as a graph that contains the cumulative *Z*-curve (the *Z* test value at each meta-analysis update), conventional level of significance, number of patients in the meta-analysis, estimated required information size, and trial sequential significance boundaries. The trial sequential significance boundaries are constructed by adjusting the thresholds for significance so that the overall risk of type 1 error is less than the desired level (usually 5%). A cumulative *Z*-curve that is greater than the trial sequential boundary is considered a statistically significant effect.

We used trial sequential analysis on the duration of analgesia. We calculated the required information size (RIS) allowing for type 1 error of 0.05, and type 2 error of 0.20, mean difference from the effect estimate from the random-effects model, and estimated variance and heterogeneity from that present in the included trials. We constructed trial sequential analysis boundaries based on the O’Brien–Fleming alpha-spending function. Trial sequential analysis software (version 0.9 Copenhagen Trial Unit, Copenhagen, Denmark) was used to perform the analysis.

### Grading of recommendations assessment, development, and evaluation (GRADE) system

We used GRADE [21] to rate the quality of evidence and the strength of recommendation of our outcome. Based on key elements including the risk of bias, inconsistency, indirectness, imprecision, and publication bias, the GRADE tool classifies the strength of synthesized evidence into four categories:

High quality: further research is very unlikely to change our confidence in the estimate of effects.

Moderate quality: further research is likely to alter the confidence in the estimate of the effect.

Low quality: further research is very likely to alter the confidence in the estimate of the effect.

Very low quality: we are very uncertain about the estimate.

## Results

**Figure 1:**
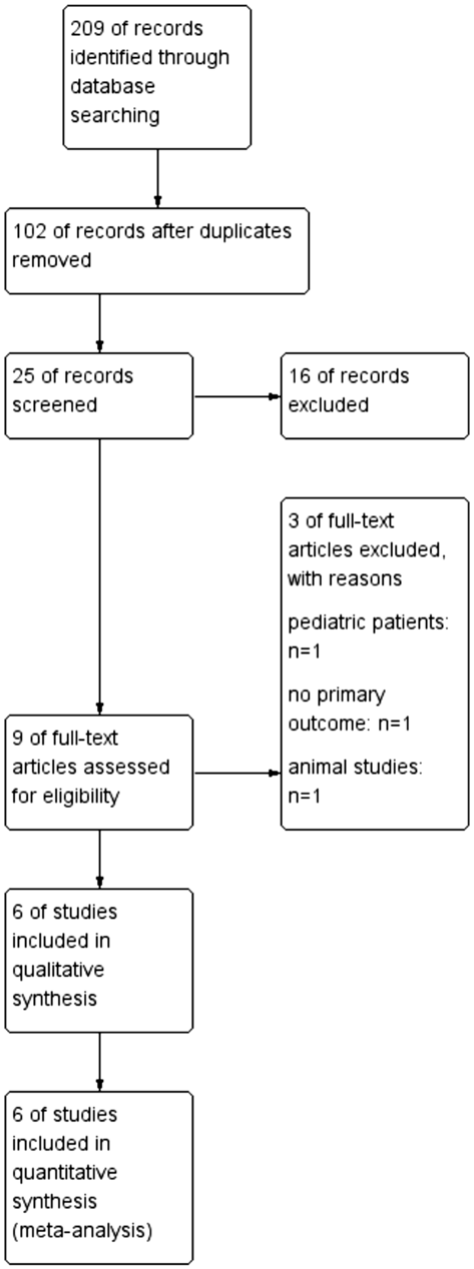
Study flow diagram (PRISMA template)

Our database search strategy retrieved 209 potentially relevant records published. Of these, a total of six full-text randomized trials were included in the final analysis. Figure 1 represents a flow diagram following the PRISMA template.

### Trial characteristics

Table 2 contains the details of the included studies. Table 3 provides quantitative results about secondary outcomes.

**Table 2:** Details of the included trials

Study	Number of patient		Type of surgery	Nerve block	Dose		Primary outcome	Other anaesthesia techniques	Postoperative analgesia
	DeM	DeA			DeM	DeA			
Sandeep Kataria	30	30	Arthroscopic shoulder surgery	Ultrasound guided interscalene block 0.5% ropivacaine 20 mL	0.5 mcg/kg	8 mg	Duration of analgesia	General anaesthesia with endotracheal intubation:fentanyl 2 mcg/kg, propofol 2 mg/kg and vecuronium 0.1 mg/kg. supplement fentanyl intra-operatively in the dose of 1 mcg/kg if there was 20% increase from the baseline parameters	PCIA (The pump was set to deliver patient-controlled boluses of 10 mcg of fentanyl, with lock-out interval of 6 min, maximum 4 h dose of fentanyl being 400 mcg)
Siamak Yaghoobi	26	25	Forearm fracture surgery	Ultrasound-guided infraclavicular brachial plexus block 28 mL lidocaine 2%	1 mcg/kg	8 mg	the time to the first requirement of analgesic supplement and the total analgesic consumption in the first 6 h postoperatively	Premedication: administration of 0.02 mg/kg midazolam and 2 µg/kg fentanyl	I.V. pethidine 25 mg,when VAS ≥ 4
Panpan zhang	20	20	Thoracoscopic pneumonectomy	Intercostal nerve block 28 mL 0.5% ropivacaine	1 mcg/kg	10 mg	Duration of analgesia	General anesthesia was induced with 0.08–0.10 mg/kg of midazolam, 0.15–0.30 mg/kg of etomidate, 2–4 µg/kg of fentanyl and 0.12 mg/kg of cisatracurium. Maintenance of anesthesia was achieved with propofol, sevoflurane, remifentanil and atracurium	received PCIA for postoperative analgesia. PCIA was administered for VAS ≥ 4 or on patient request
Julián Aliste	53	56	Upper limb surgery	Ultrasound-guided infraclavicular brachial plexus block 35 mL of lidocaine 1%–bupivacaine 0.25% with epinephrine 5 µg/mL	100 µg	5 mg	Duration of motor block	Premedication: (0.015–0.03 mg/kg of midazolam and 0.6 µg/kg of fentanyl	Not described
Myeong Jong Lee	17	17	Elective forearm and hand surgery	Ultrasound-guided axillary brachial plexus blocks with nerve stimulation 20 ml of 0.5% ropivacaine	100 µg	10 mg	The duration of the sensory block	When VAS ≥ 4 or an uncomfortable sensation developed during surgery, a 50 g bolus of fentanyl was administered intravenously. If pain persisted 5 min after administration of fentanyl, an additional 50 g fentanyl was given	Not described
Zhixin Gao	30	30	Video-assisted thoracoscopic lobectomy surgery	Ultrasound-guided erector spinae plane block 0.5% ropivacaine 30 mL	1 mcg/kg	10 mg	Postoperative PCA use during the first 72 h	General anesthesia was induced with 0.03 mg/kg midazolam, 0.5 μg/kg of sufentanil, 0.9 mg/kg Rocuronium bromide and Propofol. Maintenance of anesthesia was achieved with propofol, sevoflurane, remifentanil and cisatracurium	Sufentanil (0.1–0.2 μg/kg) and flurbiprofen (50 mg) were intravenously administered, followed by PCA pump use before the end of the surgery. PCA capacity was 250 mL and contained 7.5 μg/kg sufentanil and 250 mg flurbiprofen

*DeM* dexmedetomidine, *DeA* dexamethasone, *PCIA* patient-controlled intravenous analgesia, *VAS* visual analogue scale, *PCA* patient controlled analgesia

**Table 3:** Quantitative results

Time-to-event outcomes	Studies included	DeA		DeM		Risk ratios or weighed mean (95% CI)	*P* value for statistical significance	*P* value for heterogeneity	*I*^2^ test for heterogeneity
		*N*	Mean or *n*/*N*	*N*	Mean or *n*/*N*				
Sensory block onset (min)	[14–17]	132	12.8	133	12.66	0.4 (− 1.24, 2.04)	0.64	0.04	64%
Sensory block duration (min)	[14, 15, 17, 18]	128	656.15	126	707.52	− 9.55 (− 186.07, 166.98)	0.92	< 0.01	91%
Motor block onset (min)	[14, 16]	55	9.47	56	8.78	0.67 (0.03, 1.32)	0.04	0.97	0
Motor block duration (min)	[14, 17]	81	634.49	79	570.96	61.85 (− 178.16, 301.86)	0.61	< 0.01	96%
Fentanyl consumption (mcg)	[16, 19]	50	126.5	50	138.665	− 29.12 (− 45.18, − 13.06)	< 0.01	0.43	0
Postoperative nausea	[14–16, 18, 19]	122	7/122	123	4/123	1.6 (0.24, 10.83)	0.62	0.21	38%
Postoperative vomiting	[14–16, 18, 19]	122	8/122	123	2/123	3.89 (0.88, 17.16)	0.08	0.76	0

*DeA* dexamethasone, *min* minute, *DeM* dexmedetomidine, *CI* confidence interval

### Risk of bias assessment

**Figure 2:**
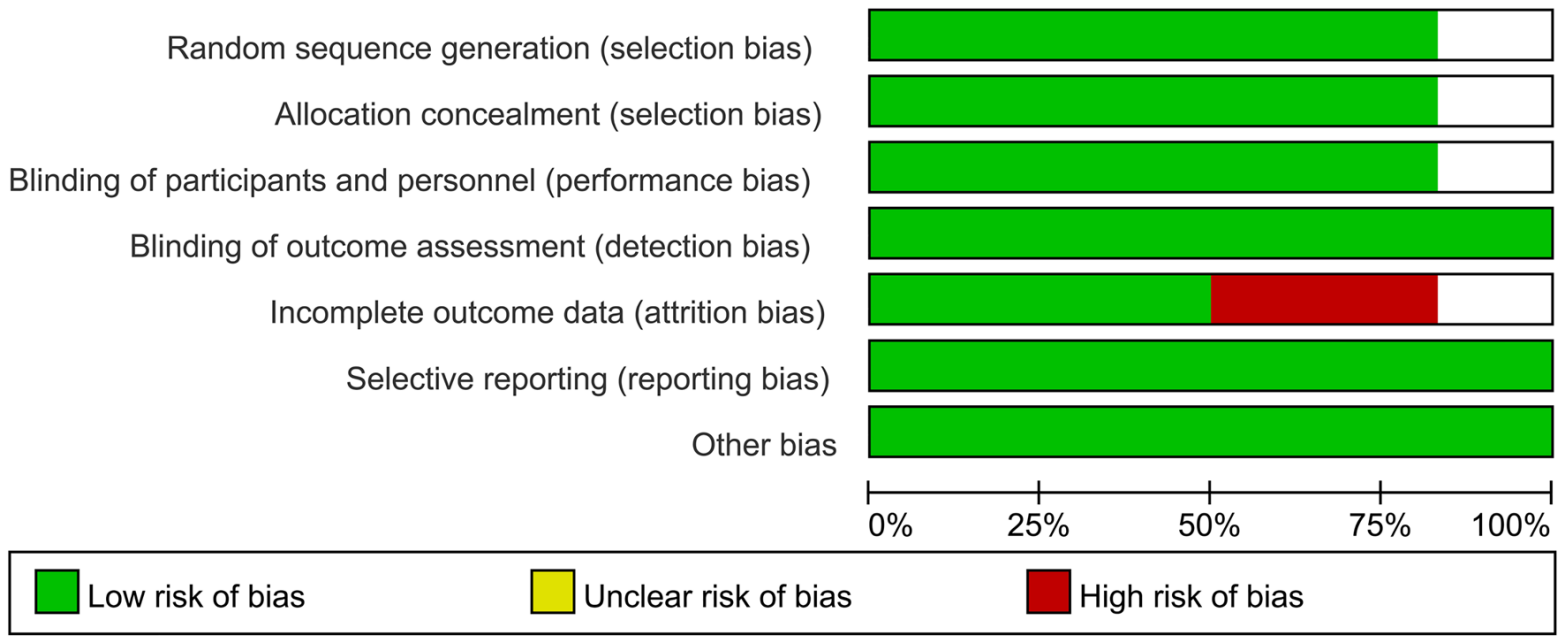
Risk of bias graph

**Figure 3:**
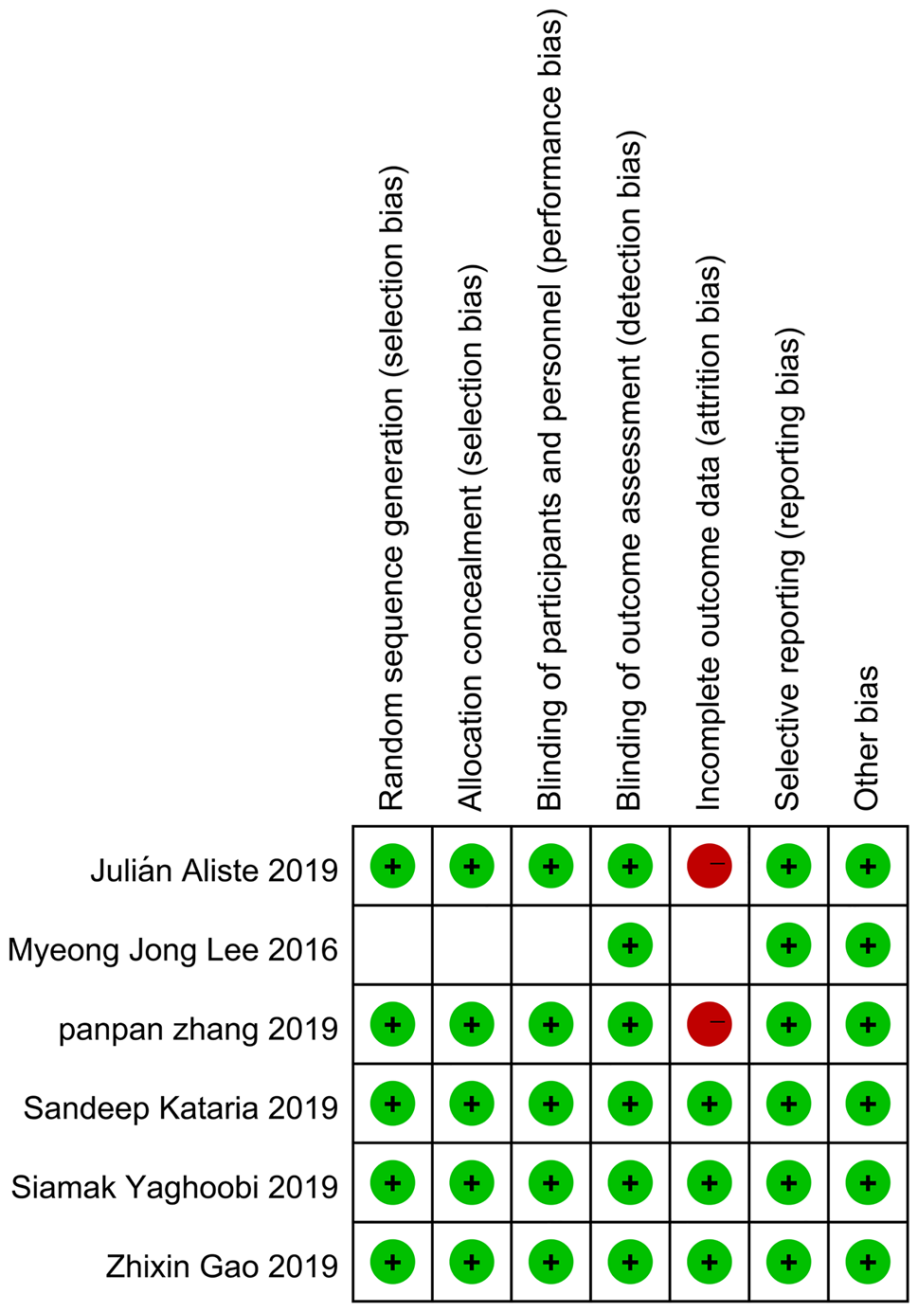
Risk of bias assessment

The methodological quality of the studies is given in Figs. 2, 3, and Table 4. We assessed five [22–26] out of six trials as low risk of bias. One trial [27] was an unclear risk due to the selection bias, performance bias, and attrition bias.

**Table 4:** The assessment judgment outcomes of RCTs

Author name (Year)	Sequence generation (Selection bias)	Allocation concealment (Selection bias)	Blinding of Participants and personnel (Performance bias)	Blinding of outcome assessors (Detection bias)	Incomplete outcome data (Attrition bias)	Selective outcome reporting (Reporting bias)	Other bias	Overall
Julián Aliste (2019)	Low risk	Low risk	Low risk	Low risk	High risk	Low risk	Low risk	Low risk
		Double blinding	Double blinding	Double blinding	Some patients lost to follow-up	The study protocol is available and all of the study’s prespecified outcomes of interest have been reported in the prespecified way	The study appears to be free of other sources of bias	
Myeong Jong Lee (2016)	Unclear risk	Unclear risk	Unclear risk	Low risk	Unclear risk	Low risk	Low risk	Unclear risk
	didn’t mention Sequence generation	didn’t mention weather about blinding	didn’t mention weather about blinding	Double blinding	Not mentioned	The study protocol is available and all of the study’s prespecified outcomes of interest have been reported in the prespecified way	The study appears to be free of other sources of bias	
Panpan Zhang (2019)	Low risk	Low risk	Low risk	Low risk	High risk	Low risk	Low risk	Low risk
		Double blinding	Double blinding	Double blinding	Some patients lost to follow-up	The study protocol is available and all of the study’s prespecified outcomes of interest have been reported in the prespecified way	The study appears to be free of other sources of bias	
Sandeep Kataria (2019)	Low risk	Low risk	Low risk	Low risk	Low risk	Low risk	Low risk	Low risk
		Double blinding	Double blinding	Double blinding	No missing outcome data or loss to follow-up	The study protocol is available and all of the study’s prespecified outcomes of interest have been reported in the prespecified way	The study appears to be free of other sources of bias	
Siamak Yaghoobi (2019)	Low risk	Low risk	Low risk	Low risk	Low risk	Low risk	Low risk	Low risk
		Double blinding	Double blinding	Double blinding	Some patients lost to follow-up	The study protocol is available and all of the study’s prespecified outcomes of interest have been reported in the prespecified way	The study appears to be free of other sources of bias	
Zhixin Gao (2019)	Low risk	Low risk	Low risk	Low risk	Low risk	Low risk	Low risk	Low risk
		Double blinding	Double blinding	Double blinding	No missing outcome data or loss to follow-up	The study protocol is available and all of the study’s prespecified outcomes of interest have been reported in the prespecified way	The study appears to be free of other sources of bias	

### Synthesis of results

#### Primary outcome: duration of analgesia

**Figure 4:**
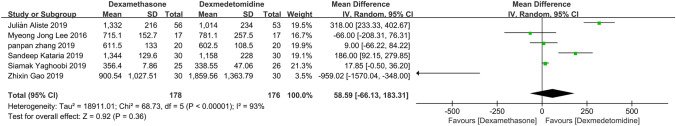
Meta-analysis: duration of analgesia (min), min, minute

Figure 4 shows the meta-analysis for the primary outcome including six trials [22–27] that had data for this outcome. When comparing peripheral dexamethasone with dexmedetomidine, the estimated duration of analgesia was 58.59 min (95%CI: −  66.13, 183.31; *P* = 0.36) longer in the peripheral dexamethasone group. But this difference did not reach statistical significance. The heterogeneity among the pooled studies was significant (*I*^2^ = 93%;* P* < 0.00001).

### Secondary outcomes

The meta-analysis is shown on the following outcomes.

### Sensory block onset

**Figure 5:**
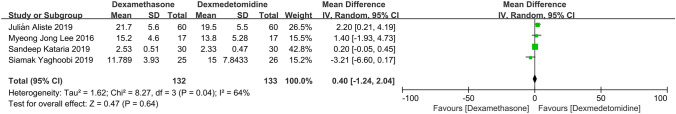
Meta-analysis: sensory block onset (min), min, minute

This outcome was reported in four studies (Fig. 5) [23–25, 27]. When comparing peripheral dexamethasone with dexmedetomidine, the estimated onset of sensory block was 0.40 min (95% CI: −  1.24, 2.04; *P* = 0.64) longer in the peripheral dexmedetomidine group. This difference was not statistically significant. The heterogeneity among the pooled studies was significant (*I*^2^ = 64%;* P* = 0.04).

### Sensory block duration

**Figure 6:**
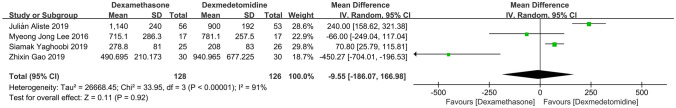
Meta-analysis: sensory block duration (min), min, minute

Four studies reported this variable (Fig. 6) [23, 24, 26, 27]. When comparing peripheral dexamethasone with dexmedetomidine, the estimated duration of the sensory block was 9.55 min (95% CI: −  186.07, 166.98; *P* = 0.92) longer in the peripheral dexmedetomidine group. This difference was not statistically significant. The heterogeneity among the pooled studies was also high (*I*^2^ = 91%; *P* ≤ 0.00001).

### Motor block onset

**Figure 7:**
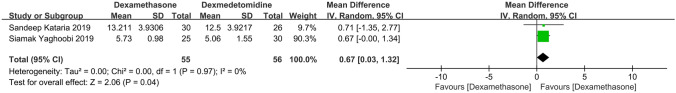
Meta-analysis: motor block onset (min), min, minute

This outcome was reported in two studies (Fig. 7) [23, 25]. When comparing peripheral dexamethasone with dexmedetomidine, the estimated onset of the motor block was 0.67 min (95% CI: 0.03, 1.32; *P* = 0.04) longer in the peripheral dexamethasone group. The heterogeneity among trials was insignificant (*I*^2^ = 0;* P* = 0.97).

### Motor block duration

**Figure 8:**
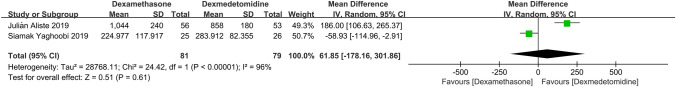
Meta-analysis: motor block duration (min), min, minute

Four studies reported this variable (Fig. 8) [23, 24]. When comparing peripheral dexamethasone with dexmedetomidine, the estimated duration of the motor block was 61.85 min (95% CI: −  178.16, 301.86; *P* = 0.61) longer in the peripheral dexamethasone group. This difference was not statistically significant. The heterogeneity among the pooled studies was significant (*I*^2^ = 96%;* P* < 0.00001).

### Analgesic consumption (fentanyl)

**Figure 9:**
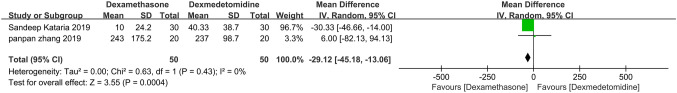
Meta-analysis: analgesic consumption (fentanyl)

This outcome was reported in two studies (Fig. 9) [22, 25]. When comparing peripheral dexamethasone with dexmedetomidine, the estimated analgesic consumption was 29.12 mcg (95% CI: −  45.18, −  13.06; *P* < 0.0004) more in the peripheral dexmedetomidine group. There was no heterogeneity among the pooled studies (*I*^2^ = 0;* P* = 0.43).

### Adverse outcomes

**Figure 10:**
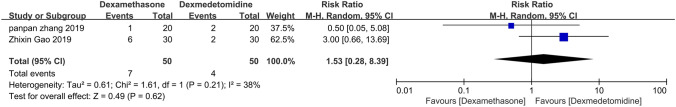
Meta-analysis: postoperative nausea

**Figure 11:**
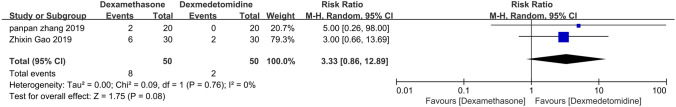
Meta-analysis: postoperative vomiting

There was no significant difference in postoperative nausea and vomiting assessed in five studies (Figs. 10, 11) [22, 23, 25–27]. Only one trial reported bradycardia [27], dizziness [22], Horner’s syndrome [25], and hoarseness of voice [25]. There were no reports of perioperative and postoperative hyperglycemia caused by dexamethasone.

### Subgroup analysis

**Figure 12:**
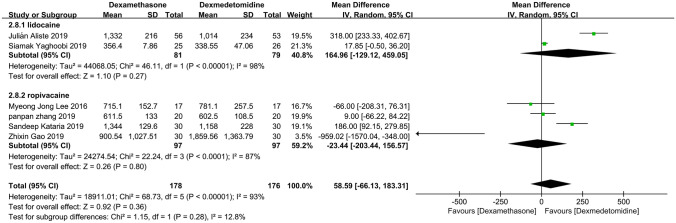
Meta-analysis: duration of analgesia (min): lidocaine versus ropivacaine subgroups, min, minute

**Figure 13:**
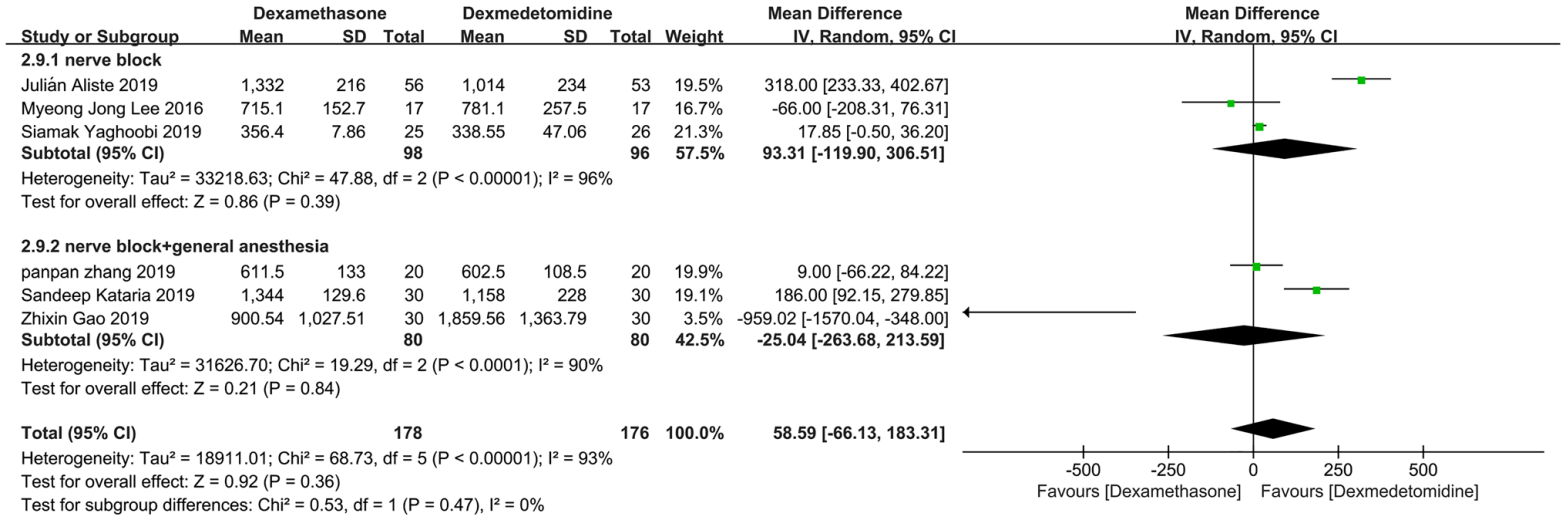
Meta-analysis: duration of analgesia (min): nerve block versus nerve block + general anesthesia subgroups, min, minute

**Figure 14:**
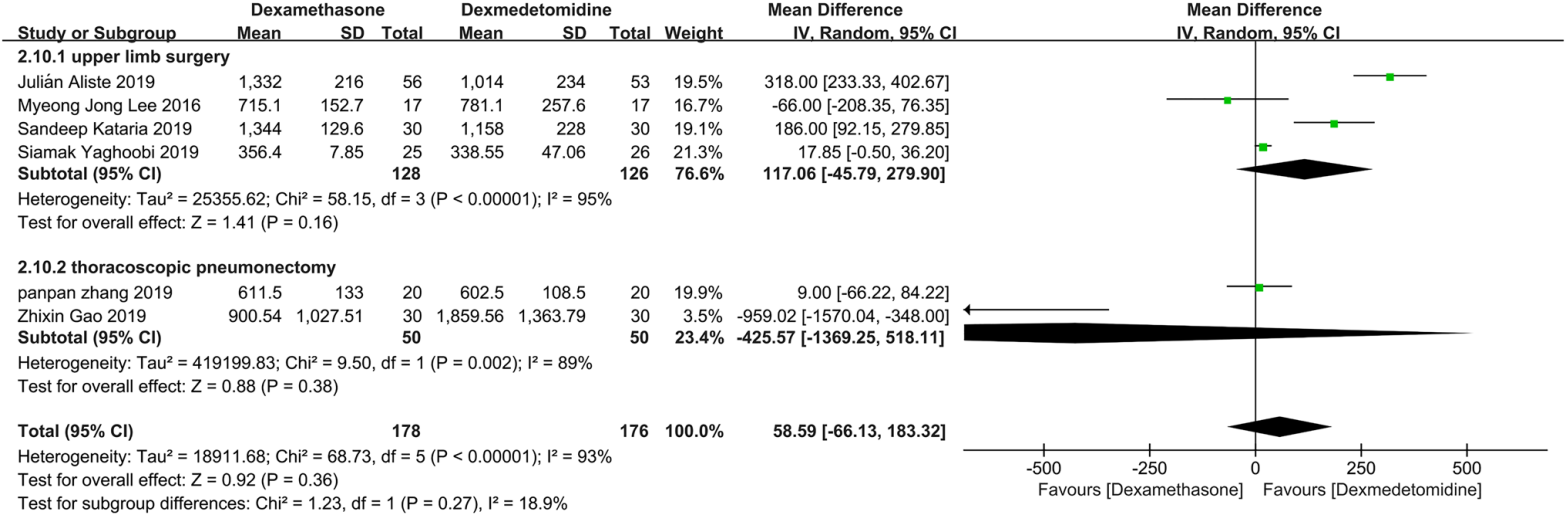
Meta-analysis: duration of analgesia (min): upper limb surgery versus thoracoscopic pneumonectomy subgroups, min, minute

Through three times subgroup analysis (Figs. 12, 13, 14), we believed there was no significant difference between the subgroups: (1) lidocaine vs ropivacaine (*P* = 0.28), (2) nerve block vs nerve block + general anesthesia (*P* = 0.47), and (3) upper limb surgery vs thoracoscopic pneumonectomy (*P* = 0.27), and the heterogeneity remained substantial.

### Sensitivity analysis

**Figure 15:**
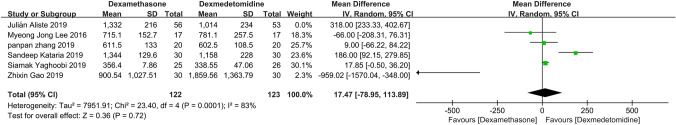
Sensitivity analysis: duration of analgesia (min), min, minute

In Fig. 15, the meta-analysis is shown after sensitivity analysis. The heterogeneity was high in our primary outcome. After removing one study [24], the *I*^2^ was lower from 93 to 83%, but there was still no statistical difference in the duration of analgesia.

### Trial sequential analysis

**Figure 16:**
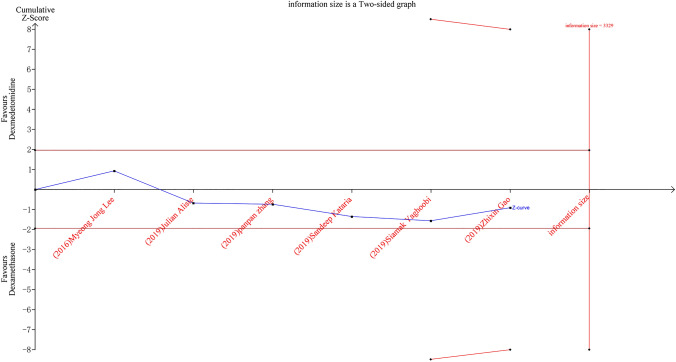
Trial-sequential analysis of six trials comparing perineural dexamethasone with dexmedetomidine for the duration of analgesia

In Fig. 16, we demonstrate that the trial sequential analysis (TSA) curve neither crosses the traditional boundary value nor the TSA boundary value, and the cumulative information size does not reach the required information size, indicating that the meta-analysis was insufficiently powered to answer the clinical question defined by the assumptions used, and more data are needed to establish this.

### Grade

We assigned the GRADE level of “low quality” to our primary outcome “duration of analgesia” (Table 5). This assessment was based on the risk of bias, demonstrated by insufficient details regarding blinding and concealment of sequence allocation and some outcomes were incomplete. Regarding the inconsistency, the *I*^2^ is high and we did not assess the risk of publication bias because of the few studies included. As a result of our assessment of the risk of bias, inconsistency, and publication bias, we down-graded the level of evidence three times, resulting in our assessment of the primary outcome being “low quality”.

**Table 5:** The grading of recommendations assessment, development and evaluation (GRADE) approach

No of studies	Design	Quality assessment					No of patients		Effects		Quality	Importance
		Risk of bias	Inconsistency	Indirectness	Imprecision	Other considerations	Analgesia	Control	Relative (95% CI)	Absolute		
Duration of analgesia (Better indicated by lower values)												
6	Randomised trials	Serious^a^	Serious^b^	No serious indirectness	No serious imprecision	None	178	176	–	MD 58.59 higher (66.13 lower to 183.31 higher)	⊕⊕ΟΟ Low	Critical
Sensory block onset (Better indicated by lower values)												
4	Randomised trials	Serious^a^	Very serious^c^	No serious indirectness	No serious imprecision	None	132	133	–	MD 0.4 higher (1.24 lower to 2.04 higher)	⊕ΟΟΟ Very Low	Important
Sensory block duration (Better indicated by lower values)												
4	Randomised trials	Serious^a^	Very serious^c^	No serious indirectness	No serious imprecision	None	128	126	–	MD 9.55 lower (186.07 lower to 166.98 higher)	⊕ΟΟΟ Very Low	Important
Motor block onset (Better indicated by lower values)												
2	Randomised trials	No serious risk of bias	No serious inconsistency	No serious indirectness	No serious imprecision	None	55	56	–	MD 0.67 higher (0.03 to 1.32 higher)	⊕⊕⊕⊕ High	Important
Motor block duration (Better indicated by lower values)												
2	Randomised trials	Serious^a^	Very serious^c^	No serious indirectness	No serious imprecision	None	81	79	–	MD 61.85 higher (178.16 lower to 301.86 higher)	⊕ΟΟΟ Very Low	Important
Analgesic consumption (Better indicated by lower values)												
2	Randomised trials	Serious^a^	No serious inconsistency	No serious indirectness	No serious imprecision	None	50	50	–	MD 29.12 lower (45.18 to 13.06 lower)	⊕⊕⊕Ο Moderate	Critical

No of studies	Design	Quality assessment					No of patients		Effect		Quality	Importance
		Risk of bias	Inconsistency	Indirectness	Imprecision	Other considerations	Adverse reaction	Control	Relative (95% CI)	Absolute		
Postoperative nausea												
2	Randomised trials	Serious^d^	No serious inconsistency	No serious indirectness	No serious imprecision	None	7/50 (14%)	4/50 (8%)	RR 1.53 (0.28 to 8.39)	42 more per 1000 (from 58 fewer to 591 more)	⊕⊕⊕Ο Moderate	Important
							–	8.3%		44 more per 1000 (from 60 fewer to 613 more)		
Postoperative vomiting												
2	Randomised trials	Serious^d^	No serious inconsistency	No serious indirectness	No serious imprecision	None	8/50 (16%)	2/50 (4%)	RR 3.33 (0.86 to 12.89)	93 more per 1000 (from 6 fewer to 476 more)	⊕⊕⊕Ο Moderate	Important
							–	3.3%		77 more per 1000 (from 5 fewer to 392 more)		

^a^Some authors did not provide sufficient details regarding blinding and concealment of sequence allocation and some outcome is incomplete.

^b^I2 statistic was high without satisfactory explanation by subgroup analysis, but it can be reduced by sensitivity analysis

^c^I2 statistic was high without satisfactory explanation by subgroup analysis

^d^Some outcome is incomplete

## Discussion

This is the first review to assess the direct effects of adjuvants, such as dexamethasone and dexmedetomidine, when applied to a regional block. Previously, Albrecht [28] conducted an indirect meta-analysis to identify the superior adjuvant by comparing dexamethasone and dexmedetomidine, and believed that dexamethasone was superior. In our meta-analysis, however, dexmedetomidine appears to have a comparable duration of analgesia with dexamethasone. A GRADE level of “low quality” was assigned to this primary outcome.

We also observed a longer duration of sensory block onset (0.40 min) and duration (9.55 min) with peripheral dexmedetomidine, and the difference was not statistically significant. The motor block was longer in the peripheral dexamethasone group, the time of onset and duration was 0.67 and 61.85 min, respectively, but the difference in motor block duration was insignificant. There was no significant difference in postoperative nausea and vomiting. It is noteworthy that dexamethasone reduced analgesic consumption (fentanyl) by 29.12 mcg compared with dexmedetomidine.

In our meta-analysis, we performed a subgroup analysis of three aspects. We found there was no significant difference between the subgroups, which indicates that the type of local anesthetic, methods of anesthesia, and type of surgery were not the reason for the high heterogeneity. Therefore, we suspect that it may be related to the dose of adjuvants and the concentration and volume of local anesthetics. However, we could not conduct a meta-regression to assess a dose–response effect because of the few studies included. We concluded from other studies that the two may be powerful influencing factors. Woo et al. carried out a randomized controlled trial and evaluated the effect of different doses of dexamethasone on the duration of single-shot interscalene brachial plexus block using ropivacaine 0.5% [29]. They concluded that dexamethasone demonstrated a dose-dependent effect on the duration of analgesia. However, Kirkham et al. conducted a meta-regression and believed 4 mg of peripheral dexamethasone represents a ceiling dose in terms of prolonging analgesia duration with very low-quality evidence [14]. The latest randomized controlled trial comparing the analgesic time of different doses of peripheral dexamethasone found 2, 5, and 8 mg of dexamethasone provide clinically equivalent sensorimotor and analgesic durations for ultrasound-guided infraclavicular block although 5 mg provided a longer analgesic duration (2.7 h) than 2 mg [30]. Therefore, the dose–effect relationship of dexamethasone is still unclear. Fredrickson et al. found that block duration is influenced by both local anesthetic volume and concentration [31].

However, we lowered the *I*^2^ of the primary outcome through sensitivity analysis. Using this, we removed one study [24] that we believed was the main source of heterogeneity. There are several explanations for the high inconsistency. First, the local anesthetic–epinephrine mixture may affect the outcome. Epinephrine itself acts as a vasoconstrictor and can prolong the duration of analgesia [32]. While Saied et al. [33] conducted an observational study and deem that epinephrine does not affect the duration of analgesia of brachial plexus block when added to ropivacaine with or without other adjuvants. Second, the number of patients in the included studies might not be adequate, although they all had considered the sample size. In addition, the included studies selected different optimal doses of the adjunct according to the different original trials.

For heterogeneity that cannot be explained by subgroup analysis and sensitivity analysis, we believed that analgesics used during peri-operation may be a critical factor. In one trial [26], patients were administered analgesia intravenously before the end of surgery, and this resulted in greater heterogeneity.

The results of our review are subject to several limitations. First, the trials included herein were small without enough power confirmed by trial sequential analysis and characterized by high levels of heterogeneity, factors that limit the clinical combinability of the source trials, and the generalizability of our results. Second, the GRADE level that we assigned to our study was only low quality for the conclusions and for the different dosages of adjuvants, we did not conduct a meta-regression to assess a dose–response effect. Also, contour-enhanced funnel plots for publication bias was limited, because the included trials were small. Finally, we did not consider the neurotoxicity of these two adjuncts, because there were no reports in our included studies. A study has demonstrated the safety of dexmedetomidine sciatic nerve block in rats [8]. Ferré et al. [34] showed that peripheral dexamethasone had a protective effect against the neural inflammation induced by bupivacaine and attenuated neural inflammation in the animal experiments. However, dexamethasone [35] and dexmedetomidine are used off-label. Even though it is widely used on an international level and has been investigated in many scientific trials, the US Food and Drug Administration does not approve dexamethasone or dexmedetomidine for peripheral administration.

In summary, dexamethasone was comparable with dexmedetomidine in terms of analgesia. However, because of the number of studies included, further comparisons are encouraged. The optimal dosages remain uncertain. Future dose-finding studies are required to elucidate the optimal dose of dexamethasone and dexmedetomidine.

## Supplementary Information

Below is the link to the electronic supplementary material.

Supplementary file1 (DOCX 21 KB)
